# School staff wellbeing: A network-based assessment of burnout

**DOI:** 10.3389/fpsyg.2022.920715

**Published:** 2022-10-05

**Authors:** Maedeh Aboutalebi Karkavandi, H. Colin Gallagher, Peng Wang, Eva Kyndt, Dean Lusher, Karen Block, Vicki McKenzie

**Affiliations:** ^1^Melbourne School of Population and Global Health, University of Melbourne, Melbourne, VIC, Australia; ^2^Centre for Transformative Innovation, Swinburne University of Technology, Melbourne, VIC, Australia; ^3^Melbourne Graduate School of Education, University of Melbourne, Melbourne, VIC, Australia

**Keywords:** burnout, social networks, teachers, social support, ERGM, brokerage

## Abstract

Burnout is commonly associated with professions that entail a high rate of close relationships with other individuals or groups. This paper explores the association between burnout and interpersonal relationships using a relational, social network framework. We collected data on advice-seeking relationships among 102 teachers and administrative staff from a secondary school in Melbourne, Australia. Burnout was measured using the Burnout Assessment Tool and we focused on four core subscales: (1) exhaustion; (2) mental distance; (3) emotional impairment; and (4) cognitive impairment. We applied a particular class of statistical model for social networks called Exponential Random Graph Models (ERGMs) to shed new light on how level of burnout relates to formation of advice relations among school staff. Results indicated that high levels of overall burnout were linked to a higher number of advice-seeking ties among school staff. Additionally, teachers who scored high in cognitive impairment (i.e., difficulties in thinking clearly and learn new things at work) tended to seek and to provide advice to a greater number of others. Finally, school staff who scored high in exhaustion (i.e., a severe loss of energy that results in feelings of both physical and mental exhaustion) tended to be sought out less as advisors to others, while those high in mental distance (i.e., psychologically distancing oneself from others) were generally less likely to seek advice from other school staff. We discuss these findings drawing on Conservation of Resource theory. Notably, our results show that burnout is not only an individual-level problem, but that burnout is associated with reduced social connectivity in specific ways that may impact on how other school staff collaborate, culminating in a staff-wide overall impact that affects how schools function.

## Introduction

Secondary school teachers are one of the professions that have the highest levels of sick leave due to burnout (García-Carmona et al., 2019). Over the last four decades there has been increasing attention paid to burnout (Sabagh et al., 2018; García-Carmona et al., 2019; Mota et al., 2021). Recently, burnout has been defined as a multidimensional work-related syndrome, indicated by exhaustion, loss of control over emotional and cognitive processes, and mental distancing (Schaufeli et al., 2019).

A wide array of research has defined burnout as a socially induced syndrome (e.g., Bakker et al., 2003; Meredith et al., 2020). Social connectedness to colleagues has long been shown to be an important determinant of burnout, even after controlling for workload (Bakker et al., 2005; Sabagh et al., 2018). What is unknown is how burnout affects interpersonal relationship and how burnout impacts school staff relationships. It is vital to answer this question, given interpersonal relations and specifically advice relations are considered an important organisational resource for learning, sharing information, and joint problem solving (Cross et al., 2001; Lazega et al., 2016). Providing advice to one another allows school staff to mobilise and exchange information, tacit knowledge, and resources (Goddard et al., 2007; Ortega et al., 2020). Consequently, the diffusion of information and knowledge can help them improve their practice, overcome challenges, and achieve their instructional goals (Vescio et al., 2008; Lane and Sweeny, 2018; Ortega et al., 2019).

To date, the literature on teacher advice relationships has overwhelmingly focused on the different opportunities networks offer to individuals because of their predispositions and their position in the network, paying little attention to the ways in which psychological traits such as burnout drive the development of networks (Borgatti and Halgin, 2011; Spillane and Kim, 2012; Siciliano, 2015). Even though it has been widely shown that these psychological traits have an impact on individuals’ (social) behaviour (Burt et al., 1998), there is little empirical evidence of on how burnout is linked to advice network structures. A joint investigation of burnout and social network structures may uncover how burnout facilitates or impedes advice seeking among school staff.

We applied an inferential social network approach known as exponential random graph models (ERGMs) to take the actual network structure into account (Lusher et al., 2013). Such models have the potential for evaluating tie formation processes while considering the complex social structure present in real-world interactions. However, these models are seldom applied in educational research (Lusher, 2011; Marion and Schreiber, 2016). Hence, this study’s contribution is twofold: from a methodological perspective, we showcased the use of a novel inferential network model in educational research. Subsequently, we provide evidence on the role of burnout in interpersonal relationships while taking the structural dependency of network ties into account.

## Theory and hypotheses

### Burnout

In a wide range of professions, burnout is currently one of the most studied consequences of stress in the workplace (Childs and Stoeber, 2012). A variety of negative outcomes have been associated with teacher burnout, including absenteeism, high attrition, and poor job performance (Martínez Ramón, 2015; Seth, 2016). Burnout was traditionally viewed as a three-dimensional phenomenon characterised by emotional exhaustion, depersonalisation and cynicism, which were measured by the Maslach Burnout Inventory (MBI) (Schaufeli et al., 2019). However, the MBI conceptualisation, psychometric properties, and practical application have been questioned (Schaufeli et al., 2019). To address this, the Burnout Assessment Tool (BAT) was developed to provide a revised conceptualisation and measurement of burnout in response to the need for updated research on burnout [For a review see Schaufeli et al. (2020)]. Schaufeli et al. (2020) define the burnout concept as both inability and unwillingness to put in effort at work. Based on the BAT operationalisation, burnout is a syndrome comprising four core components as explained by the authors: (1) exhaustion, which is a severe loss of energy accompanied by both physical (tiredness, feeling weak) and mental (feeling drained and worn out) exhaustion; (2) emotional impairment, manifested by intense emotional reactions and feeling overwhelmed by emotions; (3) cognitive impairment, which is characterised by memory problems, attention deficits, and poor cognitive performance; (4) mental distance, which indicates a psychological distance from the work and a strong reluctance or aversion toward it (Schaufeli et al., 2019).

A set of diverse risk factors can lead to chronic stress and subsequent burnout in the educational context. The possible risk factors include high workload, disruptive behaviour of students, lack of support from colleagues and supervisors, insufficient opportunities for training, promotion and professional development, low income, and deficient school and classroom facilities (Cunningham, 1983; Taris et al., 2001; Rodríguez-Mantilla and Fernández-Díaz, 2017). A recent review of antecedents of faculty burnout indicated workload contributed significantly to school staff burnout (Van Droogenbroeck et al., 2014; Sabagh et al., 2018). Furthermore, a higher workload is negatively linked to supportive relationships with colleagues at work (Van Droogenbroeck et al., 2014). Thus, to be able to draw a sound inference about the relationship between burnout and interpersonal relations at work, we controlled for workload in the current study.

### Social networks as resource passageways

In the current study, we use a social network framework to investigate the link between burnout and advice network structure. By using a social network approach this study takes the actual network structure into account and goes beyond an intra-individual focus. A social network is defined as a set of actors and the relations among them (Wasserman and Faust, 1994). In the advice network, the actors are individuals and the relations (also termed ties) between them represent advice. A fundamental concept in studies of social networks is the dependency among network ties. This assumption permits a realistic assessment of the social structure. In the current study, we use the Exponential Random Graph Models (ERGM) framework to understand what gives rise to a particular network structure. Exponential Random Graph Models (ERGM), a particular class of statistical model for social networks, is a modelling approach built on the theoretical assumption of dependency in network processes and network structures—that is, social relationships are interdependent and not independent units of analysis (Lusher et al., 2013). In ERGM models, the overall framework includes three categories of tie formation processes: network self-organisation processes, attribute-based processes, and exogenous contextual factors. Network self-organisation processes refer to the process of network ties organising themselves into patterns because the presence of some ties encourages the formation of other ties. For instance, by already being popular, an individual may attract even more network ties (i.e., preferential attachment, or the “rich get richer” in terms of network ties). Exogenous contextual factors processes refer to contextual factors that might affect tie formation (e.g., organisational hierarchy). Finally, the attribute-based process refers to how individual capacities, capabilities and predispositions are related to tie formation.

### Burnout and advice networks

Studies of organisations and schools suggest that educators utilise their social networks to inform their practice (Frank et al., 2004; Daly and Finnigan, 2012; Coburn et al., 2013a,b; Meredith et al., 2017). Furthermore, the valuable impact of advice networks has been documented on individual and organisational outcomes, including on wellbeing and job satisfaction (Flap and Völker, 2001; Edinger and Edinger, 2018; Ortega et al., 2020), employee performance at work (Podolny and Baron, 1997; Seibert et al., 2001), and organisational success (Sparrowe et al., 2001; Lazega et al., 2016). However, unlike literature that examines how structural characteristics of advice networks relates to outcomes, studies asking about individual-level antecedents are relatively rare (Nebus, 2006; Lazega et al., 2016).

We use the Conservation of Resources theory (COR) (Hobfoll, 1989) to explain how burnout relates to social network structures. We consider social networks as conduits through which resources are exchanged where actors aim to maximise resources and minimise resource loss (Kalish et al., 2015). Kalish et al. (2015) integrated COR theory with a social network framework when investigating how stress spreads through and shapes networks. The conservation of resources model is widely used to explain behaviour under stressful conditions, often with reference to occupational settings, and is one of the main theories used to understand the antecedents and consequences of burnout (e.g., Neveu, 2007; Halbesleben and Rathert, 2008). Its primary assertion is that people “strive to retain, protect, and build resources and that what is threatening them is the potential or actual loss of these valued resources” (Hobfoll, 1989, p. 516). Resources are described as “objects, states, conditions, and other things that we value” (Hobfoll, 1989, p. 514) and can be material, social and/or energetic. According to COR, actual resource loss, the perceived threat of resource loss or failure to obtain resources after significant use of resources leads to stress (Hobfoll, 1989, 1998). The COR theory has particular relevance here, as it focuses on what are termed as resource passageways where resources flow from or to people (Hobfoll, 2011). Individuals can exchange resources through network ties (e.g., advice relations) where they can identify and exchange resources that are vital for their wellbeing. Thus, a person’s network structure can influence the resources to which they have access, which might affect their level of stress and burnout. Further, stress and subsequent burnout experienced by network actors can impact the dynamics of advice ties in the network (Kalish et al., 2015).

#### Burnout and seeking advice

The literature on the impact of stress and social networks suggests that individuals who suffer from high levels of stress tend to create fewer social connections, while those who experience less stress are more expansive and flexible in forming social connections (Kalish et al., 2015; Aboutalebi Karkavandi et al., 2022). The theoretical account of the relation between stress and forming social ties comes from literature on stress such as the work of Hancock (1989). It suggests that stress is linked with physical and psychological withdrawal and consequently social withdrawal (Repetti, 1992). Additional theoretical support for the effects of stress on forming social relations such as advice-seeking ties comes from COR theory. COR theory posits that individuals need to invest resources to be able to gain new ones (Hobfoll, 2011). Asking for advice from colleagues entails a cost such as fear of rejection, losing face, and showing vulnerability (Bolger et al., 2000; Borgatti and Cross, 2003; Agneessens and Wittek, 2012; Bruk et al., 2018). School staff may seek advice as a way of coping with burnout symptoms, including limited cognitive capacity to handle classroom challenges. Schonfeld (1990, 2001) found that school staff use advice-seeking as a coping mechanism for job demands and stress. Edinger and Edinger (2018) found that teachers with larger in-school trust networks, and greater embeddedness in advice networks, reported higher job satisfaction. A high level of stress and burnout are highly correlated (Bakker et al., 2001; Boren, 2014), hence we expect to observe the same tendencies (i.e., psychological withdrawal and fear of rejection) in school staff who suffer from burnout. Thus, we argue that school staff with higher levels of burnout are likely to conserve their resources by forming fewer advice-seeking ties.

*H1. A higher score in burnout is associated with less activity in the class related advice for school staff.*

#### Burnout and advice giving

We now consider how burnout might relate to being seen as an advice-giver. Stress and burnout theories seldom focus on how the stress and burnout level of others is taken into consideration as external cues that impact interpersonal relationships (Kalish et al., 2015; Aboutalebi Karkavandi et al., 2022). Individuals may be able to assess the burnout level of their colleagues through direct verbal reports of a feeling of burnout (e.g., González-Morales et al., 2012; Meredith et al., 2020) and indirectly, by noticing fatigue, social withdrawal and cynicism (Meredith et al., 2020). Individuals use the information gathered about others’ levels of stress to make a decision about the ease of interaction with the other person (i.e., how pleasing the interaction will be) and the value of interaction (e.g., how demanding the advice relation will be) (Kalish et al., 2015). For these two reasons we expect that school staff will be less likely to seek advice from a teacher who exhibits a higher level of burnout symptoms. Advice coming from someone who is tired, withdrawn, and exhibiting poor cognitive performance is likely to be of lower quality and thus not worth the (increased) effort of interacting with that person, who is also likelier to exhibit more intense and less predictable emotional reactions (Kalish et al., 2015). Past studies that looked at the relationship between advice giving and stress indicated that individuals with a higher level of stress are less favourable sources of advice (e.g., Kalish et al., 2015; Aboutalebi Karkavandi et al., 2022). Although the empirical evidence on the relationship between advice giving and burnout is limited, the theoretical argument above allows us to formulate our hypothesis in an exploratory manner. We hypothesise that:

*H2. A higher score in burnout is associated with less popularity in the classroom-related advice for school staff.*

#### Homophily and burnout

Past studies have demonstrated that employees with a similar level of burnout tend to form relationships with one another (Bakker et al., 2001, 2003, 2005, 2006; Meredith et al., 2020). This suggests a homophily-based process in which people feel more comfortable forming relations with others who are similar, including similar psychological attributes (McPherson et al., 2001; Robins, 2015). Although a potential explanation for similarity in burnout among co-workers could be the higher workloads in specific groups, Bakker et al. (2005) showed that even after controlling for job autonomy, subjective workload, and objective workload, levels of burnout differed significantly across intensive care units.

Individuals experiencing burnout may prefer one another based on having had similar stressful experiences (Schaefer et al., 2011), and may be a source of positive reinforcement and validation for one another, by virtue of their similar attitudes (Davis, 1981) and emotions (Barsade et al., 2000). Studies of co-rumination had shown that individuals who suffer from stress, and anxiety tend to form bonds based on shared fears and worries where they validate and reinforce the negative feelings about their work environment (Haggard et al., 2011; Boren, 2014). Finally, it is possible that school staff suffering burnout tend to seek advice from one another because they become marginalised in the advice network (Schaefer et al., 2011). As we described above burnout school staff might withdraw from others (H1) and might also not be a popular source of advice for others (H2), hence they might have limited choice of whom they can seek advice from when needed. We hypothesise that:

*H3. School staff with similar levels of burnout are more likely to seek advice from each other.*

#### Burnout and brokerage

While previous hypotheses related to giving and receiving advice, burnout may also relate to being an intermediary in the network. In a general sense, a brokerage position (also variously referred to as bridging position, boundary spanning, etc.) refers to a location in the network that links people or groups who are not otherwise directly linked to one another (Long et al., 2013). Individuals in such a position are able to control the flow of information and resources across social space by bridging information gaps (“structural holes”), and coordinating collective efforts (Burt, 1992; Long et al., 2013). Importantly, though, brokers do not simply relay information; they also synthesise it. By virtue of their connection to multiple parts of the network, brokers can pick and choose the best pieces of information from multiple parts of the network, and integrate them into a new perspective (Burt, 2004). In this way, brokerage underpins many essential workplace functions, such as accessing and relaying novel information (e.g., Granovetter, 1973; Burt, 1992; Borgatti and Cross, 2003), learning how to do job tasks (e.g., Brown and Duguid, 1991; Lave and Wenger, 1991), and sharing problem-solving at work (e.g., Weick and Roberts, 1993; Moreland et al., 1996). Literature on knowledge sharing, in particular, has emphasised the significance of brokers in the facilitation of transfer of knowledge (Lomas, 2007; Penuel et al., 2009; Meyer, 2010; Neal et al., 2015, 2019).

From a COR perspective, the occupational benefits of brokerage may be seen in the development of key job resources. Job resources are “those physical, psychological, social, or organisational aspects of the job that may do any of the following: (a) be functional in achieving work goals; (b) reduce job demands and the associated physiological and psychological costs; (c) stimulate personal growth and development” (Demerouti et al., 2001, p. 501). As a result, a bridging position may buffer against burnout by opening up opportunities for job autonomy and personal development. In turn, the development of these resources may allow the individual to maintain their social position. For example, Cornwell (2009) found that older adults who maintained disconnected personal networks tended to have better physical and cognitive health. This effect is attributed to the cognitive benefit derived from having to coordinate one’s social contacts, and negotiate the flow of information back and forth.

Nevertheless, brokerage may be a double-edged sword by increasing social and work demands at the same time that it facilitates professional development. While brokerage has been robustly linked to various indicators of workplace performance (e.g., Burt, 1992, 2005), its relation to wellbeing and related variables presents a much more mixed picture. In work contexts, the impact of brokerage on wellbeing may depend heavily on the content of the network tie, with brokerage in trust networks conferring benefit to wellbeing, but instrumental brokerage coming at a cost to wellbeing [Flap and Völker, 2001; see also Podolny and Baron (1997)]. Likewise, in general social contexts, multiple scholars have found a negative association between brokerage and wellbeing (Bearman and Moody, 2004; Carboni and Gilman, 2012; Lee et al., 2019). While highly embedded individuals face less stress owing to clear and consistent expectations from their (uniform) social group (Haines et al., 2002), brokers may often face multiple sets of social expectations that can be unclear or even at odds (Podolny and Baron, 1997). Therefore, in this study, to understand brokerage as an opportunity for the development of resources, we control for job demands.

Thus, we hypothesise that:

*H4. Brokerage positions in the advice network will be negatively associated with burnout.*

## Methodology

### Participants

The participants of this study comprised teachers and administrative staff from one large, metropolitan-area secondary education school within the state of Victoria in Australia with four campuses. The school’s four campuses are highly interconnected for three reasons: 1-all campuses are geographically close, 2-they have the same leadership team, and 3-all staff members attend frequent central meetings. Data was collected mid-2021, after a prolonged period of social restrictions owing to COVID-19, including the protracted physical closure of schools in Victoria, and the institution of virtual instruction online. We invited all 140 school staff within the school to participate, with 102 responding to the survey (71% response rate). While the impact of missing data within network research is an enduring issue, this rate is considered an acceptable response rate for network research (Borgatti et al., 2006; Kossinets, 2006). The 102 participants included 63 females (61.8%), 25 males (24.5%), 1 non-binary (1%), and 13 preferred not to provide information about their gender (12.7%). The mean age of the participants was 40.98 years (*SD* = 12.35 years). The school staff average experience at the current school was 8.34 years (*SD* = 8.64 years). The majority of school staff members are teachers (*n* = 74), the rest are in leadership positions (*n* = 6), administrative positions (*n* = 1), and wellbeing support positions (*n* = 8), and 13 school staff members did not provide details about their roles.

### Procedure

Initial contact was made with school leaders. After the principal agreed to participate in the study, they allowed researchers to contact school staff. Participants were invited *via* email to an online meeting delivered *via* the Zoom video conferencing platform. In the online meeting, the researcher explained the research to the participants, provided an opportunity for questions, and invited them to take the survey *via* a secure online survey platform. Those wishing to participate were invited to take the survey at that time. All participants provided their written informed consent to participate in the study. Prior to data collection, this study was reviewed and approved by relevant ethics committees.

### Measures

#### Demographics

School staff were asked about their age, gender, years of experience at current school, and campus. These demographic characteristics were chosen as control variables based on past research on burnout prevalence (e.g., Grayson and Alvarez, 2008; Van Droogenbroeck et al., 2014; Meredith et al., 2020).

#### Burnout assessment tool

Burnout was measured using the Burnout Assessment Tool (BAT): (Schaufeli et al., 2019). Several studies have shown that BAT is a reliable and valid measure, for instance, a recent study demonstrated that BAT assessed burnout as a syndrome (second-order model) and it is equally represented in samples across six European countries and Japan (de Beer et al., 2020). Additionally, Rasch analyses demonstrated that the items of the BAT functioned well for both Belgium and the Netherlands (Hadžibajramović et al., 2020). We included four core subscales of the BAT: (1) exhaustion, eight items (item example: “Everything I do at work requires a great deal of effort”); (2) mental distance, five items (item example: “I feel a strong aversion toward my job”); (3) emotional impairment, five items (item example: “I do not recognise myself in the way I react emotionally at work.”); (4) cognitive impairment, five items (item example: “When I’m working, I have trouble concentrating”). Items were assessed on a 5-point Likert scale ranging from 1 “never” to 5 “always.” The Cronbach alpha of the exhaustion, mental distance, emotional impairment, and cognitive impairment subscales were 0.89, 0.72, 0.78, 0.87, respectively.

#### Quantitative workload inventory

Quantitative Workload Inventory (QWI) was used to determine the amount or quantity of work in a job. The QWI is a nine-item scale assessing how often each statement occurs (item example: “How often does your job require you to work very fast?”). The items are rated on a Likert scale ranging from 1 = less than once per month or never to 5 = several times per day, with a possible score from 5 to 45. High scores represent a high workload. Spector and Jex (1998) reported a good level of internal consistency with an average (coefficient alpha) of 0.82 across 15 studies. The Cronbach alpha for the current study was 0.86.

#### Network name generators

An advice-seeking network was measured by asking the following question: “Whom do you go to for class-related information.” Participants were provided with a roster of names of their co-workers at the school to select from. A matrix constituting the whole network of class-related advice relations among school staff was constructed, where a value of 1 in cell (*i, j*) indicated that, ego *i* reported seeking class-related advice from alter j, and a value of 0 indicated otherwise.

### Analysis: Exponential random graph models

Social network tie formation was examined using Exponential Random Graph Models (ERGM) for directed networks (Robins et al., 2009). ERGM are statistical models for social network structures, taking into consideration exogenous attributes of nodes or ties (Wasserman and Pattison, 1996; Snijders et al., 2006). ERGM specifications are built on a series of assumptions of conditional dependence among network ties reflecting the interdependent nature of human social activities; that is, the presence of one tie may be dependent on the existence of other ties (Robins et al., 2007; Lusher et al., 2013). ERGMS are multi-theoretical (Monge and Contractor, 2003), allowing for multiple assumptions of interdependence to be examined, each of which pertains to a social exchange principle widely described in social science. Each of these dependencies are represented by the counts of local network configurations, or graph statistics, and their parameters. The models presented in this paper are based on outputs from the MPNet software (Wang et al., 2014) which implements algorithms proposed by Snijders (2002). A more detailed tutorial discussion of ERGM with relation to social psychology may be found in Gallagher and Robins (2015), and a more general introduction to ERGM can be found in Robins et al. (2007).

The list configurations applied in the models for this paper follows the specifications proposed by Robins et al. (2001, 2009). Table 1 lists these configurations with possible interrelations. The general structural effects control for network endogenous processes, i.e., how existence of one tie may affect other ties, while the actor-relation effects represent how actor attributes affect tie formation. In our models, we focus on the interpretations of BAT related effects directly related to our hypotheses, and present other general structural and other attribute effects as additional findings.

**TABLE 1:** Exponential random graph model (ERGM) configurations.

Configurations (MPNet labels)	Interpretation
**General structural effects**	
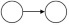 Arc	Baseline activity effect controlling for advice network density. Usually not interpreted.
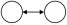 Reciprocity	The tendency for advice to be reciprocated or exchanged between a pair of nodes
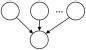 Popularity spread (AinS)	Positive effect suggests advice are centralised on a few popular advisors
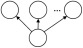 Activity spread (AoutS)	Positive effect suggests there are a few active advice seekers
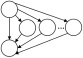 Path closure (AT-T)	Positive effect suggests network path closure result in more efficient advice flow and more cohesive closed structure.
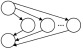 Multiple-connectivity (A2P-T)	Positive effect suggests the existences of multiple brokerage activities between advice seekers and providers
**Actor-relation effects**	
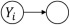 Sender	Positive effects suggest nodes with higher attribute values are more likely to seek advice
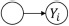 Receiver	Positive effects suggest nodes with higher attribute values are more likely to provide advice
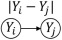 Homophily	Negative effects suggest nodes with similar attribute values, hence homophily, are more likely to seek advice from one another.
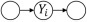 Brokerage	Positive effects suggest nodes with higher attribute values are more likely to be advice brokers

### Missing data

A 71% response rate indicates that there was some missing data. Overall, 29% of the school staff’s responses were completely missing as we did not have their demographic, burnout, or their own nominations of their social interaction partners (i.e., who they sought advice from) data. However, for these school staff with missing data, we were able to retrieve information on the social ties they were nominated in by other participating respondents. To assess non-response bias, respondents and non-respondents were compared regarding the number of times they were nominated by respondents. We ran a *t*-test on the number of ties both groups received in class-related advice network which revealed no significant difference between respondents and non-respondents, indicating that data were missing at random (MAR). This means that those people who did not complete the survey were not structurally different in network terms (i.e., they were not more isolated or more connected) to those who did complete the survey.

## Results

### Descriptive statistics

Descriptive statistics of the control and burnout variables are presented in Table 2, along with an inter-variable correlation matrix. On a scale of 1–5, school staff scored an average of 1.87 on cognitive impairment, 1.64 on emotional impairment, 2.68 on exhaustion, and 1.81 on mental distance, thus meaning on average, BAT scores can be considered average against the norms developed by Schaufeli et al. (2019). Results also show that the four dimensions of burnout were significantly correlated, with a strong correlation between mental distance and cognitive impairment.

**TABLE 2:** Descriptive statistics.

		*M*	SD	1.	2.	3.	4.	5.	6.	7.
1.	Gender	−	−							
2.	Age	40.98	12.35							
3.	Years of experience at current school	8.34	8.64	−0.05	0.54**					
4.	Workload	1.81	0.69	−0.23	0.06	0.05				
5.	Cognitive impairment	1.87	0.64	−0.06	0.01	−0.20	0.25*			
6.	Emotional impairment	1.64	0.57	0.09	0.24*	−0.07	0.18	0.60**		
7.	Exhaustion	2.68	0.88	−0.04	−0.07	−0.20	0.49**	0.65**	0.50**	
8.	Mental distance	1.81	0.69	−0.10	−0.04	−0.08	0.23*	0.70**	0.51**	0.60**

**p < 0.01, *p < 0.05.

### Exponential random graph models results

The overall ERGMs we estimated consist of endogenous network effects and node-level attribute effects. We present two ERGMs (Models A and B) throughout Tables 3–5 by three sections. Section 1 focuses on effects directly testing our hypotheses. For additional findings, Section 2 contains purely network endogenous effects, while Section 3 had other node-level attribute effects as controls.

**TABLE 3:** Actor-relation effects for aggregated burnout score (Model A) and by components (Model B).

			Model A			Model B										
			Aggregated burnout			Cognitive impairment			Emotional impairment		Exhaustion			Mental distance		
					
Effect	Configuration	Hypothesis	Para.	S.e.		Para.	S.e.		Para.	S.e.	Para.	S.e.		Para.	S.e.	
Sender	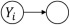	H1	0.220	0.053	*	0.412	0.078	*	0.008	0.079	−0.054	0.060		−0.140	0.068	*
Receiver	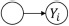	H2	0.091	0.053		0.301	0.072	*	−0.019	0.068	−0.166	0.065	*	−0.022	0.065	
Homophily	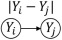	H3	−0.052	0.066		−0.048	0.080		−0.072	0.081	0.014	0.059		0.047	0.080	
Brokerage	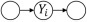	H4	−0.031	0.005	*	−0.029	0.006	*								

Statistically significant effects at 0.05 level is indicated by *.

**TABLE 4:** Network endogenous effects.

		Model A			Model B		
Effect	Configuration	Para.	S.e.		Para.	S.e.	
**Structural effects**							
Arc	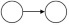	−5.550	0.436	*	−5.460	0.468	*
Reciprocity	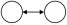	0.928	0.203	*	0.880	0.209	*
Popularity spread (AinS)	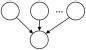	0.514	0.136	*	0.509	0.138	*
Activity spread (AoutS)	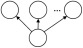	0.278	0.128	*	0.283	0.138	*
Path closure (AT-T)	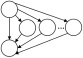	1.068	0.072	*	1.027	0.066	*
Multiple-connectivity (A2P-T)	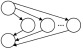	−0.103	0.018	*	−0.109	0.019	*

Statistically significant effects at 0.05 level is indicated by *.

**TABLE 5:** Other nodal attribute effects as controls.

		Model A			Model B		
Effect	Configuration	Para.	S.e.		Para.	S.e.	
**Actor-relation effects for workload**							
Sender—workload	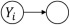	−0.001	0.008		0.000	0.008	
Receiver—workload	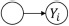	0.025	0.007	*	0.033	0.009	*
Homophily—workload	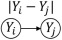	0.012	0.010		0.010	0.010	
**Actor-relation effects for years of experience at the current school**							
Sender—years of experience at the current school	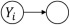	0.002	0.006		0.005	0.006	
Receiver—years of experience at the current school	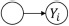	0.007	0.005		0.009	0.005	
Homophily—years of experience at the current school	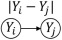	−0.013	0.006	*	−0.017	0.006	*
**Actor-relation effects for age**							
Sender—age	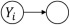	0.001	0.002		0.000	0.002	
Receiver—age	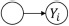	−0.002	0.002		−0.003	0.002	
Homophily—age	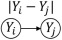	−0.001	0.003		−0.001	0.003	
**Actor-relation effects for gender (male)**							
Sender—gender (male)	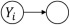	0.167	0.078	*	0.141	0.087	
Receiver—gender (male)	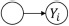	0.081	0.070		0.027	0.079	
Homophily—gender (male)	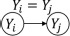	−0.191	0.106		−0.200	0.106	
Homophily—campus	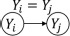	0.652	0.071	*	0.671	0.067	*
Homophily—role	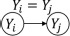	−0.002	0.087		0.008	0.096	

Statistically significant effects at 0.05 level is indicated by *.

Model A uses the total of the four scores of core symptoms of burnout (exhaustion, mental distance, cognitive impairment, and emotional impairment) as a single aggregated burnout measure. Model B treats the four scores separately, as distinct components (Schaufeli et al., 2019), each of which may affect advice tie formation differently. It is worth noting that Model B only included a brokerage effect for cognitive impairment. Not depicted here is a third model with brokerage effects (Model C) for all core symptoms as presented in the Supplementary appendix for comparison. Model B provided as adequate fit as Model C based on model GOF tests, hence Model B is a better parsimonious model. Model C also does not provide additional significant effects, while dampening the otherwise significant cognitive impairment brokerage effect in Model B.

#### Hypothesised actor-relation effects

In terms of hypothesis 1, we predicted that a higher score in burnout is associated with less advice-seeking. This hypothesis was not supported, in fact, the opposite pattern was found: higher levels of burnout were linked to more advice-seeking ties. To elaborate on more specific mechanisms that may underpin each hypothesis, we tested this hypothesis for each core symptom. There was a positive significant sender effect on advice-seeking for cognitive impairment and a significant and negative sender effect for mental distance. These results suggest that those who suffered from a high level of cognitive impairment (reduced functional capacity to regulate cognitive processes) tend to seek out advice for class-related matters from a higher number of colleagues. On the contrary, school staff who suffer from a high level of mental distance (mental withdrawal and psychological detachment) tend to seek class-related advice from fewer colleagues.

With respect to Hypothesis 2, there were mixed results. We expected that increased burnout would be associated with being sought-after for advice by fewer colleagues. While the overall burnout receiver effect was not significant (Model A), there was a significant, negative receiver effect specifically for exhaustion, indicating school staff who experienced extreme tiredness, severe and serious loss of energy tended to be less popular sources of advice among their colleagues. However, we observed the converse pattern for cognitive impairment; those who reported higher levels of confusion, difficulties in decision making and memory were nominated more often by colleagues as sources of advice.

Regarding hypothesis 3, we expected to observe a homophily effect for burnout, in which school staff with similar levels of burnout favour one another as sources of advice. However, for both overall burnout and for core symptoms, the homophily effect was not significant in either model.

Finally, regarding hypothesis 4, we expected school staff with a higher level of burnout to be less likely to act as a broker in the class-related advice network. In line with our expectation, the parameter estimates for brokerage for total burnout score was negative and significant, indicating that school staff who scored high in the burnout measure, tended not to serve as a unique bridge of advice between their colleagues. This pattern seems to be driven by cognitive impairment specifically. While a model testing brokerage across all four burnout dimensions returned no significant effects, a reduced model (model B) indicated that the parameter estimates for the brokerage for cognitive impairment was negative and significant. This suggests that school staff who experienced reduced functional capacity to regulate cognitive processes were less likely to connect their colleagues.

#### Endogenous network processes

We briefly discuss the full network model beyond our hypotheses, to identify wider social processes within the network that are important controls for the abovementioned findings. The network endogenous effects in both Models A and B control for general contextual information about the social system of advice-seeking among school staff within the school environment. We have consistent effects across the two models. Reciprocity effects were positive and significant in both models indicating high levels of mutuality in the advice-seeking network; school staff generally tends to seek advice from those who seek advice from them. Popularity spread effects were positive and significant, indicating a greater than expected variance in popularity. In other words, advice seeking ties are pointed toward a few key advisors. Activity spread effects are also positive and significant indicating some school staff was more active in seeking advice than others. The positive and significant parameter estimate for path closure (AT-T) suggests that network clustering was common in this advice network. Finally, the parameter estimate for multiple connectivity parameter was negative and significant suggesting that relatively few social bridges within the network. In other words, for school staff who are not directly connected to each other in the network, there was a tendency against having multiple, redundant mutual contacts.

#### Other actor-relation effects

With respect to network-attribute effects, for both Models A and B, there were significant homophily effects for tenure (i.e., years of experience at current school), indicating school staff with similar years of experience tend to seek advice from one another. For Model A, there is a significant and positive sender effect for male, indicating male school staff tends to seek more advice than others. Further, for both models, there were positive and significant receiver effects for workload, which shows that those who scored high in workload measures also tend to be seen as sources of advice in the network. Additionally, the homophily effect for the campus was significant, indicating school staff tend to seek advice from colleagues who work at the same campus.

## Discussion

The main aim of this study was to investigate how burnout relates to interpersonal relationships, namely advice-seeking at work for school staff. Our results point to the many ways in which burnout relates to teacher interactions. This study advances the field as there are very few studies that examine the social networks of school staff while taking an inferential social network approach, and teasing apart separate effects for the giving, receiving and brokering of advice. To the best of our knowledge, none of the past studies investigated teacher advice-seeking networks in relation to teacher burnout. Furthermore, we contribute to the field of burnout by testing our hypotheses for a single aggregated burnout measure and the four core symptoms of burnout separately. Our result indicated that four core symptoms of burnout are empirically distinct components and they are associated to advice tie formation differently. This allowed us to understand specific mechanisms that may underpin how each core symptom of burnout might play in interpersonal relationships. Our results point to the specific nature of cognitive impairment symptoms, their unique operation within a social network, and how these may differ from other components of burnout related to emotional and behavioural responses. We discuss the results of the study in terms of burnout core symptoms as follows.

The first core symptom of burnout that we discuss is cognitive impairment, manifested by reduced functional capacity to regulate cognitive processes. Our result showed that school staff who scored high in cognitive impairment, tend to seek and provide advice for class-related matters more than other school staff. As network effects are interdependent (Lusher et al., 2013), when we interpret sender and receiver effects, we must also consider the negative brokerage effect. The brokerage effect indicates that school staff with cognitive impairment are less likely to act as brokers for class-related information, which means that they were less likely to both send and receive a high level of advice. Combining three network effects (sender, receiver, brokerage) suggests that the process for sender and receiver might be separate and we may have two different profiles of the relations between advice network and cognitive impairment.

The first profile includes school staff with high cognitive impairment who rely on the advice of their colleagues to get things done. Perhaps they are seeking a lot of advice because they are overwhelmed, forgetful, and in need of a lot of help and advice. This might be a coping mechanism for burnout school staff who do not have enough cognitive capacity to deal with day-to-day classroom tasks. This result is in line with research that shows that teachers use advice-seeking as a coping mechanism for their job demands and stress (Schonfeld, 1990, 2001; Zhang et al., 2019). School staff who are demonstrating symptoms of burnout have lower cognitive capacity, hence, they might retain less knowledge. A systematic review of association between burnout and cognitive functioning suggested that burnout is linked to specific cognitive deficits (Deligkaris et al., 2014). The review of 15 English-language articles published between 2005 and 2013 revealed burnout to be associated with a decline in three main cognitive functions: executive functions, attention, and memory (Deligkaris et al., 2014).

The second profile includes school staff members who were popular sources of information for others. It is possible that they feel cognitively exhausted because lots of people are coming to them for advice. Lazega et al. (2006, 2012), studied the evolution of advice ties between judges in a commercial court. They found that network centralisation around an elite group of advisors tended to remain stable and eventually oscillate as central advisors leave or are overloaded. Cross and Thomas (2008) showed that a centralised advice network can lead to a bottleneck where few people are heavily relied on for advice. Those people may experience burnout because they are overloaded with requests for help. They might push harder in the face of endless queries, opportunities, and challenges. Such an imbalance between high job demands and insufficient resources can lead to burnout and specifically cognitive impairment (Schaufeli and Taris, 2014).

Next, our results showed that school staff members with a higher level of cognitive impairment were less likely to act as brokers for class-related information. By virtue of their social position, brokers face cognitive demands to collect and synthesise information into useful messages that can be passed along to others. As a result, only those individuals with certain abilities and skills could occupy this social position (Burt et al., 1998; Long et al., 2013). This finding also aligns specifically with research suggesting that cognitive functioning plays a particular role in pre-empting wider burnout by building up one’s job resources (Kulikowski, 2021). In particular, cognitive functioning may allow the individual to craft and maintain a relational environment that supports job tasks and professional development, thereby staving off emotional exhaustion. This study suggests that such a relational environment can be conceptualised and measured as a network environment that is underpinned by brokerage.

This result also has implications above and beyond the level of the individual, suggesting the impact of burnout at the organisational level. The association between burnout and not-brokering indicates that burned out school staff members are reducing the connectivity of the overall advice-seeking network by not filling these brokerage roles. This reduced knowledge sharing has impacts on the school staff as a whole. As such, individual burnout can reduce advice-seeking network connectivity and place a burden on other staff.

Regarding the link between mental distance and advice networks, in line with our expectations, school staff who scored a high level of mental distance (demonstrated by mental withdrawal and psychological detachment from work) seeks advice from a smaller number of their colleagues. Mental distance is a motivational aspect of burnout where individuals are not willing to be engaged with their work (Schaufeli et al., 2019). According to COR, school staff needs to invest resources such as time and energy to be able to gain new ones (Hobfoll, 2011). Those who suffer from mental distance withdraw mentally and physically from their colleagues, hence they have less interest and opportunity to invest in forming advice relations at their work environment. This finding is supported by stress literature in which physical and psychological withdrawal is associated with social withdrawal (Hancock, 1989; Repetti, 1992). Furthermore, this finding is in accordance with results from Kalish et al. (2015) and Aboutalebi Karkavandi et al. (2022) where they find that participants who were experiencing higher levels of stress were less likely to create new network ties.

Regarding the link between exhaustion and advice networks, as expected, school staff who suffered from a higher level of exhaustion were not sought-after sources of advice by their colleagues. Exhaustion shows itself as extreme tiredness and severe and serious loss of energy, these symptoms can act as external cues that affect school staff’s interpersonal relationships (Kalish et al., 2015; Aboutalebi Karkavandi et al., 2022). Individuals use the information gathered about their colleagues to make a decision about the ease of interaction with the other person (i.e., how pleasing the interaction will be) and the value of interaction (e.g., how demanding the advice relation will be) (Nebus, 2006). Colleagues of exhausted school staff might find advice relations with them demanding, possibly involving a greater cost and providing lower benefits (Kalish et al., 2015). Hence, it is not surprising that school staff who suffer from exhaustion are not getting asked about class-related information.

Regarding results for emotional impairment, we did not find any significant effects. Our result is similar to a recent study on Finnish university employees (*n* = 1,463) which showed that a sense of social belonging was not associated with emotional exhaustion (Mäkiniemi et al., 2021). Since it is not always clear to colleagues that someone is emotionally impaired, school staff need to recognise their colleagues’ breakdown and signs of emotional impairment before it impacts interpersonal relations. We propose that in the future we need longitudinal data to understand the impact of emotional impairment on advice networks (Burt et al., 1998; Long et al., 2013).

### Limitations and further research

First, when interpreting results, it is important to consider the high-stress context for this study, which occurred in the aftermath of the sudden transition to virtual instruction during the COVID-19 pandemic. During this time, school staff faced a drastic and sudden re-configuration of their job demands and were called on to translate instructional content to online platforms, and coordinate adequate resources for individual students (García-Carmona et al., 2019; Rǎducu and Stǎnculescu, 2021).

Second, the cross-sectional study nature of the data did not permit us to assess the progression of the interrelation between school staff’s burnout and advice-seeking behaviour, nor determine a causal link between burnout and advice network structure. We cannot draw a firm conclusion, for example, whether cognitive impairment leads to more advice seeking, a high level of advice seeking leads to cognitive impairment, or there is a bi-directional effect between advice seeking and cognitive impairment. Hence, for future work, we recommend examinations of advice networks and burnout at more than one-time point to be able to unpack the causal link between burnout and advice relations. It would be useful to test at the start, middle and end of the school year. In this case, we can unpack further if the link between burnout components and advice seeking and giving behaviour stays the same or changes over time. For instance, we could investigate whether school staff who suffer from a high level of cognitive impairment at the start of the school year and seek and give advice more than average continue to do so, or do they give up on their interpersonal relations by the end of the year?

Third, from a theoretical perspective, the similarity between colleagues could arise because of selection and/or social influence. Longitudinal data would provide an opportunity to untangle selection versus influence effects. We can answer whether school staff select school staff with a similar level of burnout (social selection), or whether colleagues who have frequent interactions become similar overtime because they share their negative feelings and emotions and their relations act as a conduit to pass on the feeling of burnout. Alternatively, both mechanisms may be occurring and, similar to Van Zalk et al. (2011), individuals with burnout tend to choose colleagues who with burnout, and over time they influenced each other to become more burned out.

## Conclusion

Taken together, our findings do not suggest that burnout leads to a simple blanket withdrawal from one’s participation in a professional setting. Furthermore, it was only for those with increased exhaustion, in particular, that we observe the hypothesised avoidance by colleagues. Instead, burnout is associated with not being in a position to synthesise and relay information from person to person. Combining results regarding brokerage with our results in terms of exhausted staff members being less selected as advisors, we conclude that burnout not only affects individuals suffering from burnout but also reduces the social interaction of others around them. That is, because one teacher is burned out, another teacher does not approach them for advice. Individual burnout thus affects the common pool of resources for advice-seeking. This suggests we need to consider the impacts of burnout beyond the individual in question, and the possibility of cascading effects thereby reducing the capacity for shared leadership and support.

## Data availability statement

The raw data supporting the conclusions of this article will be made available by the authors, without undue reservation.

## Ethics statement

The studies involving human participants were reviewed and approved by Swinburne University’s Human Research Ethics and the Department of Education and Training Ethics Committees. The patients/participants provided their written informed consent to participate in this study.

## Author contributions

MA contributed to the research design, data collection, data analysis, and write up. HG contributed to the research design, data collection, and write up. PW contributed to the data analysis and write up. EK contributed to the research design. DL contributed to the research design and write up. KB and VM contributed to the research design and data collection. All authors contributed to the article and approved the submitted version.
