# Supplementary material for: School staff wellbeing: A network-based assessment of burnout
Source: Front Psychol. 2022 Oct 5;13:920715. doi: 10.3389/fpsyg.2022.920715 (PMC9580495; doi:10.3389/fpsyg.2022.920715)
Supplement: Supplementary file 1 [file Table_1.pdf]

Appendix

We present Model C here to show that by including brokerage effects for all four components of the burn out measures, Model C is a more complex but not more adequate model. At the same time, by including more brokerage effects, Model C dilute the significant brokerage effect for cognitive impairment as observed in Model B.

Effect	Configuration	*para.*	*s.e.*	
**Structural effects**				
Arc	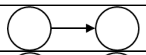	-5.525	0.433	*
Reciprocity	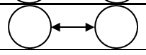	0.881	0.206	*
Popularity spread (AinS)	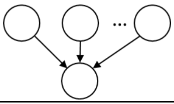	0.513	0.136	*
Activity spread (AoutS)	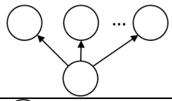	0.293	0.137	*
Path closure (AT-T)	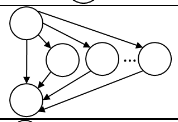	1.041	0.071	*
Multiple-connectivity (A2P-T)	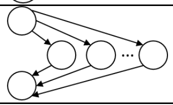	-0.105	0.017	*
**Actor-relation effects for cognitive impairment**				
Sender— cognitive Impairment	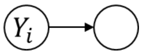	0.341	0.107	*
Receiver— cognitive impairment	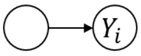	0.221	0.104	*
Homophily— cognitive impairment	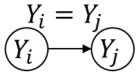	-0.036	0.082	
Brokerage — cognitive Impairment	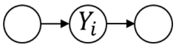	-0.015	0.013	
**Actor-relation effects for emotional impairment**				
Sender— emotional impairment	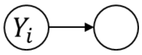	0.035	0.094	
Receiver— emotional impairment	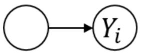	0.025	0.095	
Homophily— emotional impairment	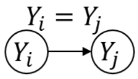	-0.081	0.086	
Brokerage — emotional Impairment	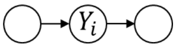	-0.007	0.011	
**Actor-relation effects for exhaustion**				
Sender — exhaustion	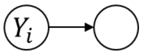	-0.028	0.078	
Receiver — exhaustion	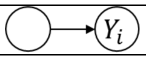	-0.140	0.076	
Homophily — exhaustion	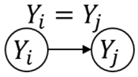	0.009	0.064	
Brokerage — exhaustion	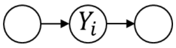	-0.005	0.009	
**Actor-relation effects for mental distance**				
Sender — mental distance	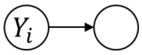	-0.120	0.105	

Receiver — mental distance	○ → (Y_i)	-0.005	0.083	
Homophily — mental distance	$Y_i = Y_j$<br>(Y_i) → (Y_j)	0.046	0.078	
Brokerage — mental distance	○ → (Y_i) → ○	-0.004	0.011	
**Actor-relation effects for workload**				
Sender — workload	(Y_i) → ○	0.001	0.009	
Receiver — workload	○ → (Y_i)	0.033	0.009	*
Homophily — workload	$Y_i = Y_j$<br>(Y_i) → (Y_j)	0.011	0.010	
**Actor-relation effects for years of experience at the current school**				
Sender — years of experience at the current school	(Y_i) → ○	0.006	0.006	
Receiver — years of experience at the current school	○ → (Y_i)	0.009	0.006	
Homophily — years of experience at the current school	$Y_i = Y_j$<br>(Y_i) → (Y_j)	-0.018	0.007	*
**Actor-relation effects for age**				
Sender — age	(Y_i) → ○	0.000	0.003	
Receiver — age	○ → (Y_i)	-0.003	0.002	
Homophily — age	$Y_i = Y_j$<br>(Y_i) → (Y_j)	-0.001	0.003	
**Actor-relation effects for gender (male)**				
Sender — gender (male)	(Y_i) → ○	0.135	0.093	
Receiver — gender (male)	○ → (Y_i)	0.029	0.085	
Homophily — gender (male)	$Y_i = Y_j$<br>(Y_i) → (Y_j)	-0.197	0.116	
**Actor-relation effects for gender**				
Homophily — campus	$Y_i = Y_j$<br>(Y_i) → (Y_j)	0.664	0.068	*
Homophily — role	$Y_i = Y_j$<br>(Y_i) → (Y_j)	0.019	0.095	
